# Synthesis of New Modified with Rhodamine B Peptides for Antiviral Protection of Textile Materials

**DOI:** 10.3390/molecules26216608

**Published:** 2021-10-31

**Authors:** Petar Todorov, Stela Georgieva, Desislava Staneva, Petia Peneva, Petar Grozdanov, Ivanka Nikolova, Ivo Grabchev

**Affiliations:** 1 Department of Organic Chemistry, University of Chemical Technology and Metallurgy, 1756 Sofia, Bulgaria; petenceto_2@abv.bg; 2 Department of Analytical Chemistry, University of Chemical Technology and Metallurgy, 1756 Sofia, Bulgaria; st.georgieva@uctm.edu; 3 Department of Textile and Leathers, University of Chemical Technology and Metallurgy, 1756 Sofia, Bulgaria; grabcheva@mail.bg; 4 The Stephan Angeloff Institute of Microbiology, Bulgarian Academy of Sciences, 1113 Sofia, Bulgaria; grozdanov@microbio.bas.bg (P.G.); inikolova@microbio.bas.bg (I.N.); 5 Faculty of Medicine, Sofia University “St. Kl. Ohridski”, 1407 Sofia, Bulgaria; i.grabchev@chem.uni-sofia.bg

**Keywords:** rhodamine-peptides, hemorphins, cotton fabric, absorbance, emission, virucidal effect, antiviral activity

## Abstract

Here we report on the synthesis and characterization of three new N-modified analogues of hemorphin-4 with rhodamine B. Modified with chloroacetyl, chloride cotton fabric has been dyed and color coordinates of the obtained textile materials were determined. Antiviral and virucidal activities of both the peptide-rhodamine B compounds and the dyed textile material were studied. Basic physicochemical properties (acid-base behavior, solvent influence, kinetics) related to the elucidation of structural activity of the new modified peptides based on their steric open/closed ring effect were studied. The obtained results lead to the conclusion that in protic solvent with change in pH of the environment, direct control over the dyeing of textiles can be achieved. Both the new hybrid peptide compounds and the modification of functionalized textile materials with these bioactive hemorphins showed virucidal activity against the human respiratory syncytial virus (HRSV-S2) and human adenovirus serotype 5 (HAdV-5) for different time intervals (30 and 60 min) and the most active compound was Rh-3.

## 1. Introduction

The xanthene dyes are widely used in medicinal and bioorganic chemistry as biologically active compounds (either alone or conjugated). These compounds possess potent antiviral activity against a human foreskin fibroblast (vesicular stomatitis virus) and enhance the antiviral activity of xanthene derivatives from 8- to 15-fold [1]. Rhodamine B derivatives represent an important tool for studies of more complex biochemical processes and activities. Such kind of fluorescence-based probes of bioactive molecules possess desirable features; they have an excellent spectral characteristics and relatively facile syntheses [2,3]. Moreover, rhodamine B and its derivatives do not have a cytotoxic effect [4]. It is inexpensive, resistant under a variety of reaction conditions, can be covalently linked to bioactive molecules such as peptides, and has suitable spectral properties in terms of absorption and fluorescence wavelength. Rhodamine B is a lipophilic cation belonging to the family of xanthenes, and its derivatives are widely employed as fluorophore probes [3,5]. It is known that they are sensitive as fluorescent turn-on compounds. The only drawback to the use of rhodamine B is the formation of a spirolactam compound, which is non-fluorescent and cannot be used for all fluorescent microscopic applications [6]. Recently, a new class of rhodamine derivatives displaying a broad-spectrum antiviral activity against different enveloped viruses including an HSV-2 acyclovir resistant strain has been reported [7]. Unfortunately, many viral infections are the cause of death worldwide, and still now there are no efficient antiviral drugs or vaccines for a large number of viruses, and this represents a great challenge especially for emerging and re-emerging viral diseases [7]. Moreover, the peptides and peptide-conjugated molecules, having a propensity to interact with membrane interfaces, might exert broad antiviral activity against enveloped viruses. [8].

The peptides can be useful as substituents of the proteins, especially when site-specific modification of the required protein is difficult or impractical. Peptides are sufficiently small molecules which can be easily modified in the laboratory using standard synthetic protocols and solid-phase peptide (SPPS) methods. The large part of peptides can play role of ligands, since they contain a significant number of precisely located functional groups and amino acid residues, which possess high-affinity and specific interactions with a target receptor. This is usually harder to achieve with small molecules [9,10]. The peptide scaffolds allow the introduction of fluorophores in their structure while retaining the biological activity, which can increase the possibilities for the design of fluorescent sensors and their applications in biochemistry and medicine [11,12]. This kind of fluorophore molecule is incorporated into or appended to a known peptide sequence that has high affinity for the target protein. The presence of amino acid residues that have both aromatic moiety and hydrophobic nature, such as Trp, Phe, and Tyr, favor protein binding. Replacement of this residue with fluorophore provides a significant change in protein binding and a correspondingly strong enhancement of the fluorescence signal [9].

Hemorphins are endogenous peptides, belonging to the family of atypical opioid peptides, released during the sequential cleavage of hemoglobin proteins [13,14,15]. Hemorphins have been shown to exhibit diverse therapeutic effects in both human and animal models [16,17,18]. Hemorphin-4 (Tyr-Pro-Trp-Thr) is a member of the hemorphins family, an endogenous nonclassical opioid peptides derived from hemoglobin. There is evidence that these kinds of natural or synthetic tetrapeptides exert opioid activities in vivo and, therefore, may play an important physiological role [19]. Our previously investigations have demonstrated that not only the position of modification, but also the nature of the incorporated group, lead to significant changes in the peptide activity and affinity [20,21,22,23,24,25]. Recently, the rhodamine B-labelled arginine-rich peptide has been designed as the heparin bioreceptor to construct a highly sensitive and selective fluorescent biosensor for heparin detection [26]. However, there are no data in the literature concerning rhodamine conjugated hemorphin analogues.

A recent study has reported that the hemorphins bind with high affinity to angiotensin-converting enzyme (ACE) [27]. ACE2 is expressed in nearly all human organs in varying degrees. Both SARS-CoV-2 and SARS-CoV enter host cells via the angiotensin-converting enzyme 2 (ACE2) receptor [28]. Thus, targeting the ACE2 receptor of host cells can block the entry of the virus into the cell, thereby protecting the host from viral infection and pandemic disease COVID-19 [29].

Different methods of textile materials functionalization exist in order to achieve biologically-active effects. Most of these methods/reagents are controversial for humans and the environment due to inorganic salts, phenols and thiophenols, antibiotics, formaldehyde derivatives, etc. which are used during production [30]. Therefore, the development of new methods has to fulfil the requirements of being safe for both human health and the environment. In conjunction with such functionalization, there is a question concerning to which extent the normal skin flora of healthy people will be destroyed by action of an antimicrobial compound [31]. Therefore, the increasing tendency of research is seen where the functionalization is performed by the use of non-toxic, biodegradable, and environmentally-friendly reagents. Currently, the peptides are the candidate therapeutic agents that offer selectivity and specificity, and low levels of side effects. The great advantage of peptides against viruses consists in the reduced possibility of developing resistance during the treatment [32]. Cotton has widespread use in textiles and in healthcare environments. Peptides can be simply adsorbed on cotton through electrostatic interactions but without covalent bonding they are easily lost. Functionalization of cotton can impart suitable groups for covalent bonding of different polymers and ensure durability of modification during use [33].

Herein we report on the synthesis and characterization of new N-modified analogues of hemorphin-4 with rhodamine B. We have also investigated the modification of the functionalized cotton fabric with the new hybrid peptide compounds. The potential antiviral and virucidal activities of both peptides and textiles material have also been studied.

## 2. Results and Discussion

### 2.1. Chemistry

We have synthesized and characterized new rhodamine B-conjugated hemorphin-4 analogues as a potential sensitive fluorescent probe for color, antiviral, and virucidal activity of textile materials. These peptides contain different aliphatic amino acid residue and differed by the increased number of methylene group (from one to three) between rhodamine B moiety to the N-side and the amino acid scaffold of natural hemorphin-4. The aim of this study was to determine evidence of the significance of different amino alkyl residues of newly synthesized hybrid compounds for their physicochemical properties and to investigate their structurally-related properties and potential textile applications using different methods. We have also explored an approach to the structural features of pH-dependent equilibrium between the spirolactam form and the ring-opened form of these peptides, the potential antiviral and virucidal activities of both of the new hybrid peptide molecules, and the modification of functionalized cotton fabrics with these bioactive hemorphins.

**Figure 1:**
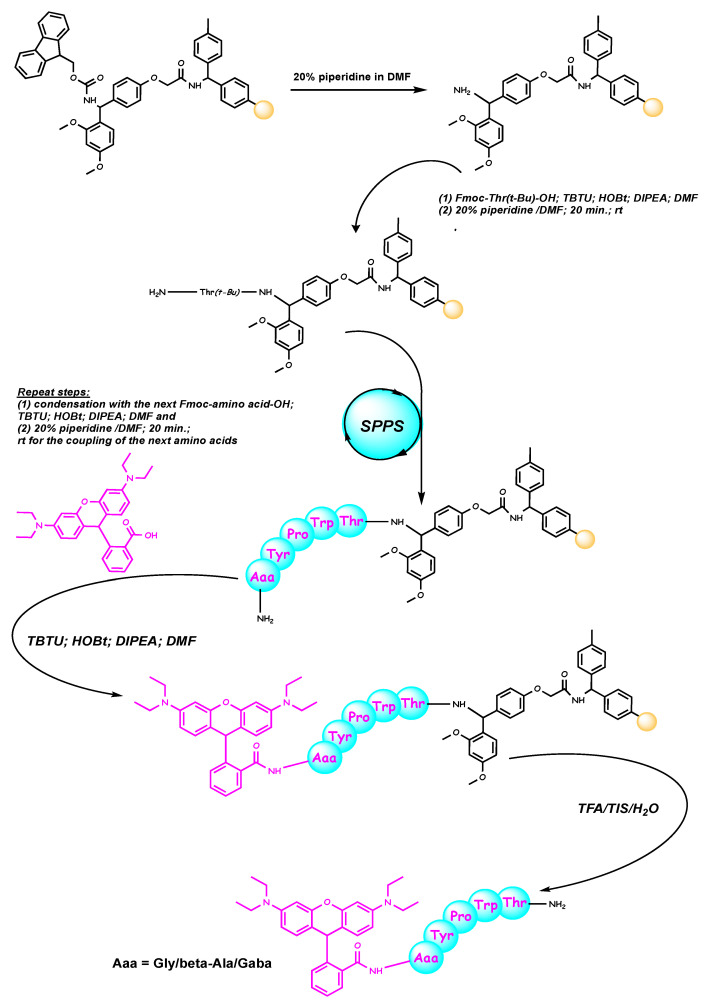
Schematic representation of the solid phase synthesis of the new hybrid peptides.

RhodamineB-Gly-Tyr-Pro-Trp-Thr-NH_2_ (Rh-1), rhodamineB-β-Ala-Tyr-Pro-Trp- Thr-NH_2_ (Rh-2), and rhodamineB-γ-Abu-Tyr-Pro-Trp-Thr-NH_2_ (Rh-3) were efficiently prepared via solid-phase peptide synthesis (SPPS) using Fmoc (9-fluorenylmethoxy- carbonyl) chemistry. This strategy, based on the reaction between rhodamine-B with the N-terminal amino group of the hemorphin-4 analogues, was applied directly to the resin. The synthetic route is summarized in Figure 1. In order to achieve peptide bond formation and to improve the efficiency of peptide synthesis, the organic compounds such as TBTU (2-(1H-benzotriazole-1-yl)-1,1,3,3-tetramethylaminium tetrafluoroborate) and HOBt (hydroxybenzotriazole) as coupling reagents, and DIPEA (N,N-diisopropylethyl- amine) as an organic base were added to reaction media in each step. After cleavage of the product from Rink-Amide MBHA Resin, the compounds were purified from crude product by semi-preparative HPLC with a C18 column. The Mass spectra confirmed the spirolactam form of peptides formation.

The handling of the amino acidic scaffold can be regarded as a potentially powerful tool in both bioorganic and medicinal chemistry investigations and the development of new drugs and materials [34].

### 2.2. Physicochemical Characterizations

#### Determination of Physicochemical Constants

**Figure 2:**
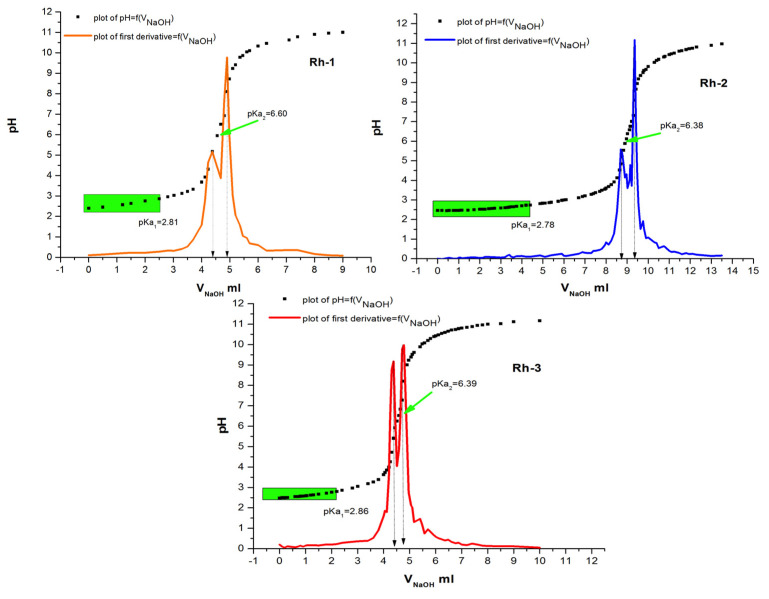
Plot of pH vs. V_NaOH_ and first derivatives graphics of 20 µmoL of Rh-1, Rh-2, and Rh-3, respectively.

**Figure 3:**
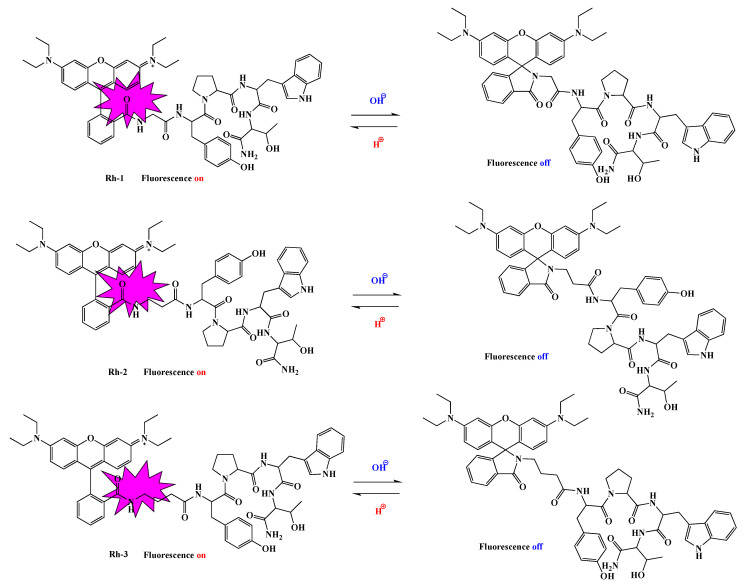
pH-dependent equilibrium between the spirolactam form and the ring-opened form of rhodamine B-conjugated hemorphin-4 analogues.

The values of the dissociation constants of the test compounds were determined using potentiometric titration. The calculated values are important for the direct application of the compounds in the process of their bonding to the textile material. It is known that one of the most important physical and chemical factors of micro-and macromolecules is the value of the acid dissociation constant (pK), used to determine the type of individual protonated and unprotonated forms of compounds and to measure the strength of acids and bases. Basically, the physicochemical properties of the compounds depend on the pH of the medium. This parameter is an important factor in the dyeing process and the interaction of the dye with the textile material. After data processing from the titration, the dissociation constants of the compounds were determined by the Hassenbach equation [23,24]. From Figure 2 it can be seen that rhodamines are referred to as diprotic acids, dissociating gradually. The calculated values of pKa and pI are summarized in Table 1. The values of the dissociation constants of the three rhodamine derivatives are approximately equal but can still be arranged in the following sequence: pK_1_(Rh-2) < pK_1_(Rh-1) < pK_1_(Rh-3). Since the pKa of Rhodamine B defines the equilibrium between the spirocyclic form and the ring-opened form, it can be reasoned that the pKa values of rhodamines could be modulated by introducing different amino acid residues of the peptide chain. pH-dependent equilibrium between the spirolactam form and the ring-opened form of Rh-1, Rh-2, and Rh-3 are shown in Figure 3. As mentioned, compounds 1 and 2 have a slightly lower value than 3, which may be due to the closer proximity of the hydroxy group of Tyr to the spirocyclic carbon, where the steric effect may be more pronounced. The calculated values of the isoelectric points show that in a weakly acidic medium (pH~4) the compounds will have zero charges (neutrality of the molecule) and, accordingly, insolubility which was taken into account at the textile dyeing.

### 2.3. Spectral Characterizations

**Figure 4:**
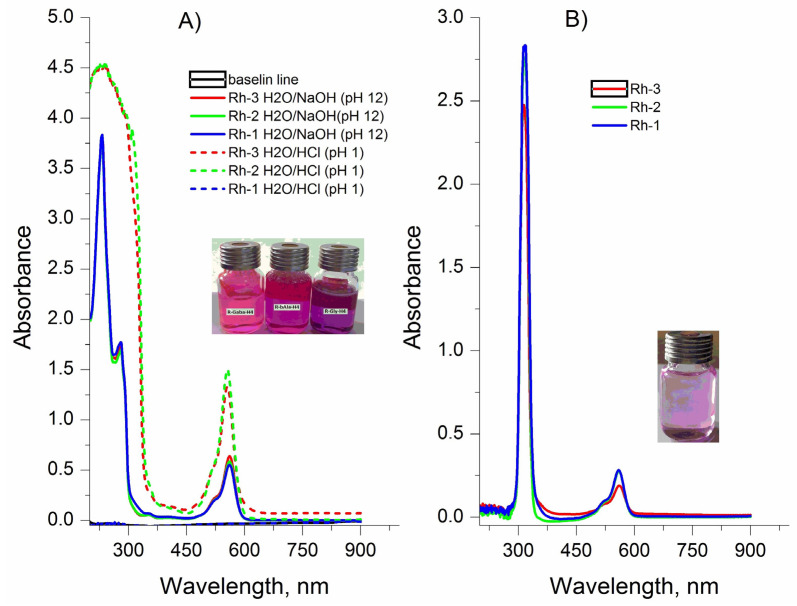
UV-Vis spectrum of Rhodamine derivatives at: (**A**) equal concentration of the compounds and different pH of solutions. Absorbance of the solution was measured against water; (**B**) solutions of Rh-1, Rh-2 and Rh-3 in triethylamine with equal concentration of the compounds. Absorbance of the solution was measured against triethylamine.

**Figure 5:**
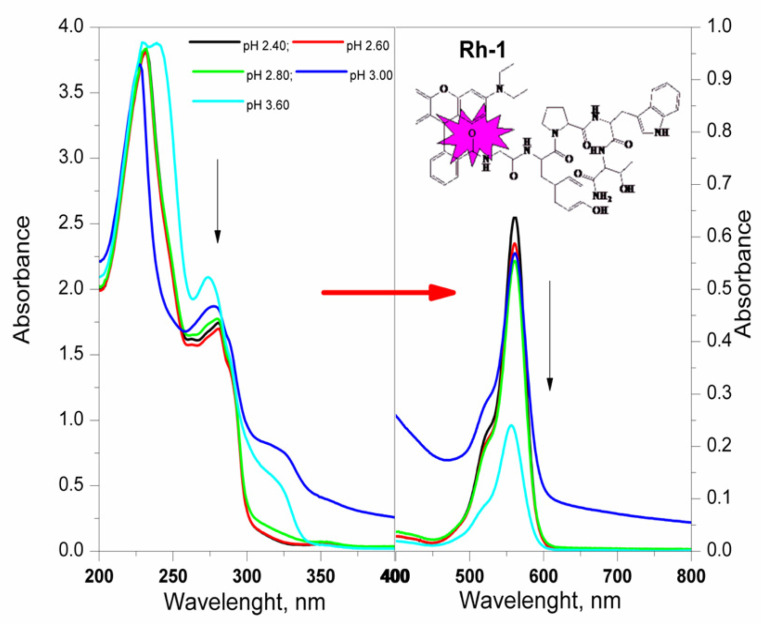
Uv-Vis spectrum of Rh-1 (4.09 × 10^−5^ mol L^−1^) at different pH.

The new compounds were characterized by FT-IR, UV-Vis, and fluorescence spectroscopy in two types of solvents (water (polar protic solvent) and triethylamine (polar aprotic solvent)) in order to investigate changes in the color of the solution directly related to the structure of the compounds. The spectra related to the absorption of electromagnetic radiation of the rhodamine derivatives are given in Figure 4. Two well-formed absorption peaks with different intensity in both used solvents, localized at λmax ≈230, ≈278 nm and 561 nm in the water solutions and λmax ≈ 300 nm and 565 nm of solution of triethylamine, respectively, can be seen (Figure 4). Basically, theπ→π∗ transitions of the > C=O in the peptide bonds is occurred by UV absorption of the peptide molecule in the range 180 to 230 nm. The aromatic side-chains of indole of Trp, phenol rings of Tyr, and rhodamine are primarily responsible for absorption of the π-electron systems of aromatic groups in the range of ≈300 nm [35,36]. The absorption bands occurring at this wavelength are equally intense due to the same number of peptide bonds and the concentration of analyzed solutions. The studied dependence on the pH of the water medium showed that the colors of the aqueous solutions remain unchanged from the strongly acidic medium in which they dissolve to the strongly alkaline one. There is a visible change in the hue of the solutions for the individual compounds, but the difference in the wavelength at which the compounds absorb in the visible region is minimal (2–5 nm). The studied dependence on the pH of the water medium showed that the colors of the aqueous solutions remain unchanged from the strongly acidic medium to the strongly alkaline one. The absorption peak intensity increased three-fold with the decrease of pH values from 12 to 1 (Figure 4 and Figure 5).

**Figure 6:**
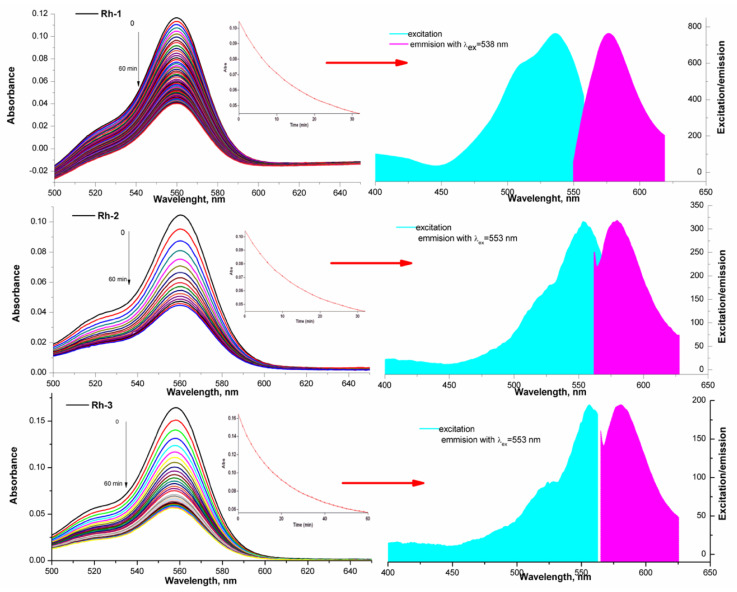
Uv-Vis kinetic plot of the first-order reaction between ring-opened and formed of the spirolactam form at equal concentration of Rh-1, Rh-2, and Rh-3 (at 25 °C) and corresponding fluorescence spectra in aprotic solvent (triethylamine). Rh-1 λ_abs_ (nm): 565, λ_em_ (nm): 589, Stokes shift (cm^−1^): 721; Rh-2 λ_abs_ (nm): 561, λ_em_ (nm): 593, Stokes shift (cm^−1^): 962; Rh-3 λ_abs_ (nm): 559, λ_em_ (nm): 592, Stokes shift (cm^−1^): 997.

**Figure 7:**
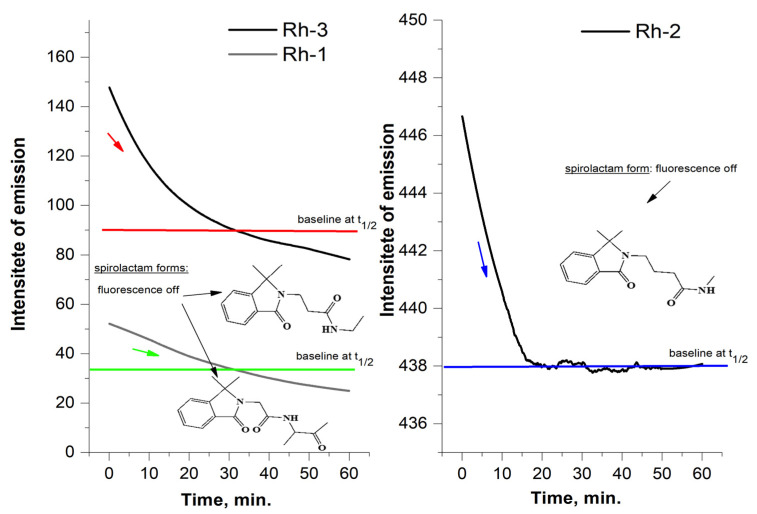
Fluorescence kinetic plot of the first-order reaction between ring-opened and formed of the spirolactam form at equal concentration of Rh-1, Rh-2 and Rh-3 and 25 °C.

A similar property of rhodamine-related peptides was observed by Meng-Chan Xia et al. [37]. Color stability was observed in the aqueous solutions of the compounds, although the intensity of the absorption maxima depended on the pH of the medium. The molar extinction coefficients in the long-wavelength absorption maximum are, respectively, Rh-3 (ε = 1.83 × 10^4^ cm.mol.L^−1^); Rh-1 (ε = 2.04 × 10^4^ cm.mol.L^−1^); and Rh-2 (ε = 2.16 × 10^4^ cm.mol.L^−1^). The behavior of the compounds in an aprotic solution of triethylamine is different. For about 6–120 min, a visible fading of the solution is observed until the complete disappearance of the color. This is the due to the observed steric effect in the molecules of the compounds associated with the case of spirolactam ring opening. This provoked our interest in studying, in addition, the influence of the environment on the structural changes of the compounds. The effect of the peptide substituent on the ring opening/closing time is clearly visible with studying the kinetics of the process. For this purpose, the spectra of the solution were taken at the initial moment, immediately after its preparation and subsequently on every 5–10 min. Figure 6 shows the decrease in the intensity of the absorption peak. The kinetic lines were taken at the same time interval and by fluorescence (Figure 7). The observed small Stock shift is typical for rhodamine dyes. It is characteristic and of the newly synthesized rhodamine B-conjugated hemorphin-4 analogues. However, the increase in the spacer length between chromophore and peptides leads to a minor enhancement of Stock shift due to the changes in molecule mobility [5,38].

From a thermodynamic point of view, the opening/closing reaction of the spirolactam ring is a first-order reaction [36]. The kinetic law with respect to the exhaustion of one form relative to the other is ln (C_0_)/C) = k.t, i.e., the dependence ln C = f (t, min) is linear. Since the concentration of the colored form of the compound is proportional to the absorption (from UV-Vis) and the emission intensity (in fluorescence analysis) in this equation, the concentration can be replaced by the corresponding physical quantity according to the literature [39]. The rate of conversion of the ring-opened form to spirolactam was quantified by calculating the value of the rate constant of the process and half the time using data from the emission spectra of the compounds. The rate constant k was calculated from the slope of plot of the left side of Equation (2) versus time and the obtained value are summarized in Table 1. The value of the rate constant (k) was used to calculate and the τ1/2—half time or this is the time for which the current concentration (C) of the ring-opened form decreases twice with respect to the initial concentration of (C_0_), i.e., when C = C_0/2_, then t = τ_1/2_. For a first-order reaction, the value was calculated by the equation: τ_1/2_ = ln2/k (Table 1) [39]. Moreover, Rh-1 had its maximum absorption band at 565 nm and strongest fluorescence emission at 589 nm in triethylamine solution (Figure 6) and the slowest spirolactam form formation effect (k = 4.6 × 10^−2^ s^−1^, Table 1) associated with solution discoloration (Figure 7). In the other compounds, the emission intensities and the value of the rate constant increase in proportion to the increase in the number of methylene groups in the peptide chain (Figure 6 and Figure 7). Stokes shift is an important feature that shows the differences between the structure of the fluorophore in the ground S_0_ state and in the first excited state S_1_ and was also calculated for the rhodamine peptide derivatives (Figure 6). The Stokes shift of the test compounds are in the range of 721 and 997 cm^−1^, which is consistent with rhodamine derivatives known in the literature [40].

**Figure 8:**
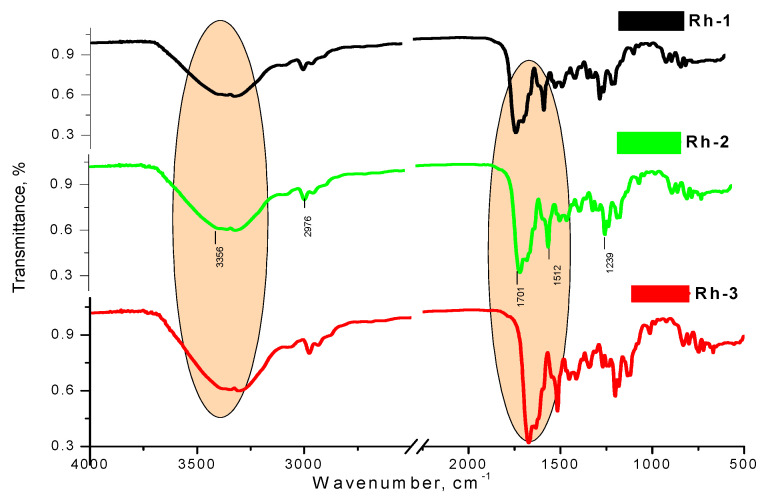
IR-spectra of the rhodamine-peptide derivatives.

The IR spectrum of studied compounds were recorded in KBr tablet (KBr, cm^−1^) and shown main characteristic bands, localized at as follow: 3356 (N-H stretching vibration (ν_NH_)), 1701 (s)—NCO (amide) stretching and 1678–1722 cm^–1^—a high-intensity peak of ν_C=O_; 1511–1528 cm^–1^ (δ_NH_) (Figure 8). As can be seen, the absorption lines in addition prove the functional groups belonging to the structure of the compounds (Figure 8).

### 2.4. Color Characterisation of Cotton Fabrics

**Figure 9:**
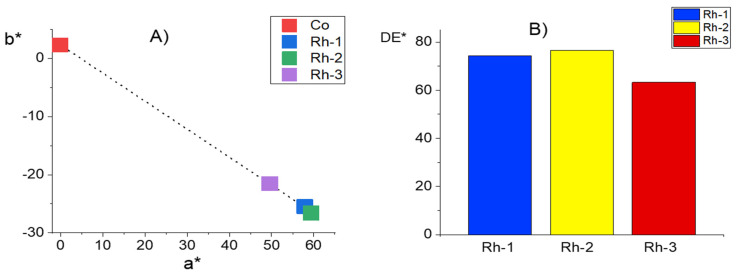
Comparison of the colour coordinates of cotton fabric (Co) and fabrics dyed with Rh-1, Rh-2, and Rh-3: *(***A**) color characterization with a* and b* coordinates; *(***B**) color difference DE*.

CIELab coordinates were used to distinguish the difference in the color of three cotton fabrics dyed with rhodamine-peptides. Figure 9 shows the change in the value of a* and b* of pristine cotton fabric and the functionalized with chloroacetyl chloride fabrics treated with peptides Rh-1, Rh-2 or Rh-3. The untreated cotton fabric has a white color in daylight with coordinates a* and b*, approximately equal to zero. For the dyed samples, the color coordinates as an absolute value increase in the order Rh-3 < Rh-1 < Rh-2 and correspond to a red-blue color. The color difference of the fabric compared with untreated fabric changes in the same order. This is in agreement with molar absorption coefficient of the new rhodamine-peptide samples. For all compounds, this coefficient is higher than 10^4^ which demonstrate a good coloring ability. A better result has been obtained with Rh-2.

**Figure 10:**
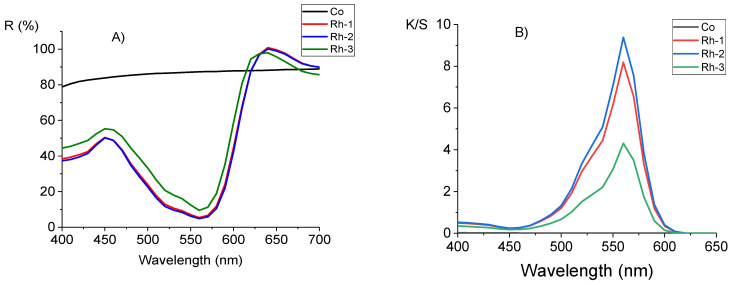
Comparison of cotton fabric (Co) and fabrics dyed with Rh-1, Rh-2, and Rh-3: (**A**) reflection spectra; (**B**) K/S values in function of wavelength.

Figure 10A shows the reflection spectra of the initial cotton fabric and after it dyeing with Rh-1, Rh-2, and Rh-3. The minimum reflectivity (R%) occurs at about 560 nm, where rhodamine peptides have the maximum absorption. As the dyes are fluorescent, there is also a band with a maximum wavelength of 640 nm [41]. Both the minimum and maximum are more pronounced for samples Rh-1 and Rh-2 compared to those of Rh-3. Figure 10B shows the relation of K/S values with the wavelength. The maximum absorbance for the three dyes is 560 nm. The K/S values give information for the dye quantity on the fabric and its behavior on the textile substrate [42]. It can be seen that the concentrations of the fixed dyes under dyeing conditions are different. Figure 10B shows the relation of K/S values with the wavelength. The maximum absorbance for the three dyes is 560 nm. The K/S values give information for the dye quantity on the fabric and its behavior on the textile substrate. It can be seen that the concentrations of the fixed dyes under dyeing conditions are different. The K/S value is the highest for Rh-2, but the value of Rh-1 is closed to it. The value of Rh-3 is twice as small as Rh-2. The reason for this can be the difference in molecular mass of this rhodamine-peptide and its different behavior in solution.

### 2.5. Fastness Testing

**Figure 11:**
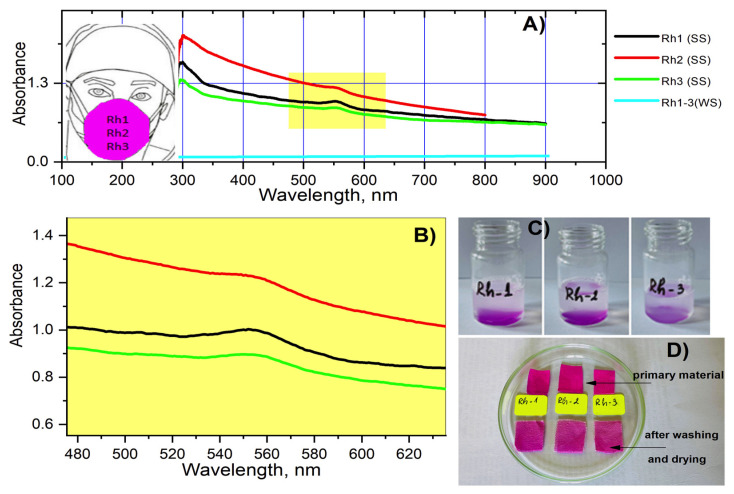
UV-Vis (zero) (**A**) and zoomed at 560 nm (**B**) spectra of soap (SS) and water (WS) solutions obtained after washing the materials; soap solutions immediately after washing the materials (**C**); compare the color of the material before and after washing with soap and water. The photos were taken after drying the materials (**D**).

The stability of the textile material after dyeing with the new antiviral agents was also studied. In order to evaluate the rebinding of the compounds when washing the dyed textile material with soap and water, the spectra of the obtained soap solutions were taken following the washing procedure. When washing the materials with water only, no difference in the spectrum of the solutions after soaking the materials was observed (Figure 11). Studies have shown that in all compounds immediately after washing with soap, the color of the fabric is slightly affected and after drying there is a visible fading of the materials (Figure 11). As can be seen from the figure most strongly absorbs the solution of Rh-2 which can be attributed to a lower binding affinity with the textile material and lower resistance to washing in an alkaline environment. However, no difference was observed in the color of the textiles when soaking the materials in the washing solution (water and soap) for 1 and 24 h.

### 2.6. Virological Activity

Antimicrobial peptides are able to inhibit many pathogens, including Gram-negative and Gram-positive bacteria and fungi. Additionally, some antimicrobial peptides have been shown to have anticancer or antiviral activity, such as indolicidin that has activity toward HIV [43]. Peptides with antiviral activity against influenza can be divided into three main groups. First, entry blocker peptides such as a FluPep that interact with influenza hemagglutinin block its binding to host cells and prevent viral fusion. Second, several peptides display virucidal activity, disrupting viral envelopes (e.g., Melittin). Finally, a third set of peptides interacts with the viral polymerase complex and act as viral replication inhibitors such as PB1 derived peptides [44].

Rhodamine B-conjugated hemorphin-4 analogues demonstrated a virucidal effect against human respiratory syncytial virus (HRSV-S2) and human adenovirus serotype 5 (HAdV-5) for different time intervals (30 and 60 min). The most active is Rh-3 peptide analogue, which is an analogue of hemorphin-4 containing a rhodamine B residue at the N-terminus and a hydrophobic -γ-Abu-Tyr-Pro-Trp-Thr-CONH_2_ amino acid sequence of the peptide molecule. The difference between others two compounds is only between one amino acid residues and in particular this is the methylene group (from one to three). Compound Rh-3 showed higher virucidal activity against HRSV-S2 at 60 min, unlike compound Rh-2 which is more active at 30 min. (Table 2). All of the peptides did not show any virucidal activity against HAdV-5 in both 30- and 60-min intervals. Perhaps this is due to the structure of HAdV-5 (non enveloped virus) which do not have lipid bilayer envelope, thus making them more resistant to chemicals.

For a more in-depth study of the virucidal effect against both HRSV-S2 and HAdV-5, cotton fabrics dyed with rhodamine-peptides have been also studied. Compared to rhodamine B-conjugated peptides, the virucidal effect of the textile materials was lower. (Table 3). In this case, the virucidal effect of cotton fabrics is due to their direct contact with viruses. The good retention of the tested peptides to the fabric surface does not allow their easy release and reaction with the viruses in solution, which explains the low virucidal activity of the fabrics to the tested viruses.

**Figure 12:**
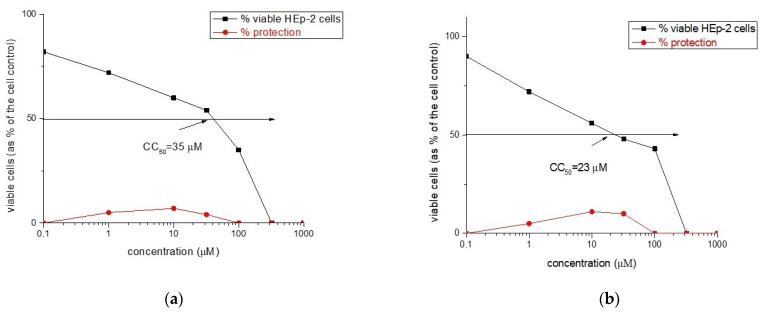
Antiviral activity curve (in red) and cytotoxicity curve (in black) of: (**a**) Rh-1 and (**b**) Rh-2.

**Figure 13:**
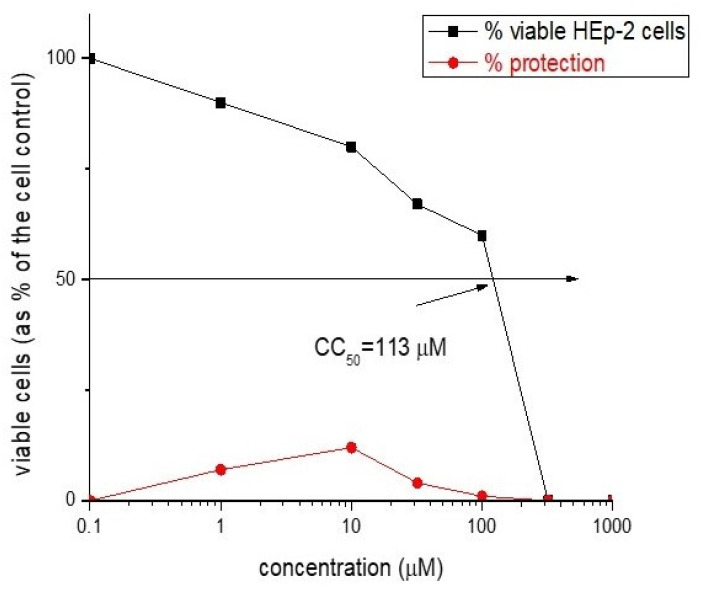
Antiviral activity curve (in red) and cytotoxicity curve (in black) activity of Rh-3.

The newly synthesized rhodamine B-conjugated compounds shown weak virucidal activity. Rh-3 is more potent as opposed to Rh-1 and Rh-2 (see Figure 12 and Figure 13).

Our experimental data suggest that in the new hybrid peptide compounds containing a rhodamine B residue, not only the position of the modification but also the nature and length of the amino acid sequence leads to significant changes in peptide activity and affinity. The results suggest that incorporation of different amino acids at the N-terminus of the hemorphin-4 scaffold deserve further evaluation in antiviral and virucidal effects.

The cytotoxicity data and antiviral activity of the compounds against human respiratory syncytial virus (HRSV-S2) and human adenovirus C serotype 5 (HAdV-5) in HEp-2 cell culture are shown in Table 4.

## 3. Materials and Methods

### 3.1. Synthesis of the Peptides (Rh-1, Rh-2, and Rh-3)

All reagents and solvents were analytical or HPLC grade and were bought from Fluka or Merck, and used without further purification. The protected amino acids and Fmoc (9-fluorenylmethoxycarbonyl)-Rink Amide MBHA (4-methylbenzhydrylamine) Resin were purchased from Iris Biotech (Germany). The 3-functional amino acids were embedded as follows: Tyr—as N^α^-Fmoc-Tyr(tBu)-OH, Thr—as N^α^-Fmoc-Thr(t-Bu)-OH, and Trp—as N^α^-Fmoc-Trp(Boc)-OH.

The solid-phase peptide synthesis by Fmoc chemistry was used to obtain new rhodamine B-conjugated hemorphin-4 analogues. The Fmoc-Rink-Amide MBHA resin was used as solid phase carrier to obtain the C-terminal amide derivatives and 2-(1H-benzotriazole-1-yl)-1,1,3,3-tetramethylaminium tetrafluoroborate (TBTU) was used as a coupling reagent. The coupling reactions were performed using for amino acid/TBTU/HOBt/DIPEA/resin a molar ratio of 3/2.9/3/6/1, in a 1:1 mixture of DMF/DCM. A 20% piperidine solution in N,N-dimethylformamide (DMF) was used to remove the Fmoc group at every step. After each reaction step, the resin was washed with DMF (3 × 1 min), isopropyl alcohol (3 × 1 min) and CH_2_Cl_2_ (3 × 1 min). The coupling and deprotection reactions were checked by the Kaiser test [45,46]. The cleavage of the synthesized peptide from the resin was done, using a mixture of 95% trifluoroacetic acid (TFA), 2.5% triisopropylsilan (TIS) and 2.5% water. The peptide was obtained as a filtrate in TFA and precipitated with cold, dry ether. The precipitate was filtered, dissolved in water and lyophilized to yield the compounds as a powder. The crude peptides were dissolved in H_2_O and acetonitrile was added until complete dissolving was observed. The peptides were obtained as white powders with a purity of >97% as determined by analytical HPLC. The structures were confirmed by high-resolution electrospray mass spectrometry. The purity of the peptides was monitored on a reversed-phase high-performance liquid chromatography (RP-HPLC), column: SymmetryShield^TM^ RP-18, 3.5**µ**, (50 × 4.6 mm), flow: 1 mL/min, H_2_O (0.1% TFA)/CH_3_CN (0.1% TFA), gradient 0→100% (45 min) and 100% (5 min). The crude peptides were purified using semi-preparative HPLC, column XBridge^TM^ Prep C18 10 **µ**m (10 × 250 mm), flow: 5 mL/min, H_2_O (0.1% TFA)/CH_3_CN (0.1% TFA), gradient 20→100% (50 min). The analytical data for the synthesized peptides (Supplementary Materials) prepared were as follows: compound Rh-1 *t_R_* 25.90 min, 1045.5062 calculated [M + H^+^], 1046.5051 observed [M + H^+^]; compound Rh-2 *t_R_* 26.60 min, 1059.5218 calculated [M + H^+^], 1060.5421 observed [M + H^+^]; compound Rh-3 *t_R_* 27.20 min, 1073.5375 calculated [M + H^+^], 1074.5581 observed [M + H^+^]. The rhodamine B-conjugated hemorphin-4 analogues were checked by optical rotation in methanol (*c* = 0.01) at 20 °C, [α]_589_^20^ as follows: compound Rh-1—40°, compound Rh-2—34°, compound Rh-3—48°.

### 3.2. Physicochemical Characterization

#### 3.2.1. Spectral Measurements

“Varian-Cary” Spectrophotometer has been used for UV-vis spectrophotometric measurements. The fluorescence spectra were recorded on a Perkin Elmer LS55 spectrophotometer at the same concentrations. The concentrations of the compounds in the triethylamine and double distilled water spectral solutions are as follows: Rh-1: C = 4.09 × 10^−5^ mol L^−1^; Rh-2: C = 3.63 × 10^−5^ mol L^−1^; Rh-3: C = 3.98 × 10^−5^ mol L**^−^**^1^. All used reagent were analytical grade. The kinetic data for the degree of open/closed of spirolactam ring were obtained according to Equations (1) and (2) and the rate constant k was fitted by Equation (2) (open→close of the ring) taking into account first-order of the reactions [39]:

ln (I_0_/I_t_) = k.t (1)

(2) $$\ln\left(\frac{I_{\infty }-I_{0}}{I_{\infty }-I_{t}}\right)=k.t$$

where (I_0_), (I_t_), and (I_∞_) refer to the signal proportional to the initial-, time t-, and final intensity of emission, respectively.

The IR spectrum was recorded in potassium bromide (KBr) pellet with a Varian 660 FTIR spectrophotometer the spectra in the 4000–500 cm**^−^**^1^ range using a Fourier Transform Infrared Spectroscopy (FT-IR). The sample was scanned 256 times with a resolution of 2 cm**^−^**^1^. The molecular mass and purity of the compound was confirmed by high-resolution electrospray mass spectrometry on a Q Exactive high-resolution mass spectrometer (Thermo Fisher Scientific Inc., San Jose, CA, USA) equipped with TurboFlow TM Transcend chromatography system (Thermo Fisher Scientific Inc., San Jose, CA, USA) and heated electrospray ionization (HESI II) source. Data acquisition and processing were done by XCalibur**^®^** 2.4 software (Thermo Fisher Scientific Inc., San Jose, CA, USA). The instrumental parameters were as follows: Spray Voltage—4.0 KV, Sheath Gas—30 AU, Auxiliary Gas—12 AU, Capillary Temperature—300 °C, Spare Gas—3 AU, Heater Temperature—300 °C. Full scan experiments were carried out in a range of 120–2000 *m*/*z* at 140,000 resolution.

Optical rotations were recorded on an MCP200 modular circular polarimeter (Anton Paar Opto Tec GmbH, Seelze, Germany).

#### 3.2.2. Potentiometric Titration

A digital pH-meter (Jenway) was used for potentiometric titrations of the compounds in order to determinate pKa and pI values. For each titration, 5.00 mL from stock water solutions of the rhodamine derivatives were titrated with standardized base (0.0112 mol L^−1^NaOH). Data (volume of titrant vs. pH) were processed by Origin8Pro software for determination of pKa of the sample.

### 3.3. Cotton Fabric Modification and Characterization

#### 3.3.1. Functionalization of Cotton Fabric with Chloroacetyl Chloride

Cotton fabric (140 g/m^2^) was modified with 10% (*v*/*v*) chloroacetyl chloride (ClCOCH_2_Cl) in N,N-dimethylformamide at 25 °C for 2 h. The solution was of liquor to goods ratio of 20:1. Then the fabric has been removed from the solution, washed thoroughly with water, and dried in the open air [47].

#### 3.3.2. Dyeing of Functionalized Cotton Fabric with Rh-1, Rh-2, and Rh-3

The dyeing solution of rhodamine-peptide derivatives were prepared by dissolving each dye in DMF and water (the ratio 1:14.5). The modified with chloroacetyl chloride cotton fabric was impregnated with relevant dye solution (2.0 owf %) at a liquor to goods ratio 5:1, and then dried at room temperature for 30 min and at 50 °C for 60 min. The colored cotton fabric has been washed thoroughly with water.

#### 3.3.3. Characterization of Cotton Fabrics

The color coordinates (L* a* b*) and the reflectance spectra (R%) of dyed cotton fabrics have been determined by using Datacolor Spectraflash SF300 spectrophotometer (Datacolor, NJ, USA) and Micromatch 2000**^®^** software. The samples were measured under illuminant D65 using the 10° standard observer. A non-treated cotton fabric has been used for the color difference quantification [48].

For the assessment of the depth of color and the obtained shade from rhodamine-peptides the Kubelka–Munk theory and Equation (3) has been applied.

K/S = (1 − R_λ_)^2^/2 · R_λ_ (3)

where K is the light absorption coefficient, S is the light scattering coefficient, and R_λ_ is the reflectance of a dyed fabric.

#### 3.3.4. Fastness Testing

The dyed fabrics were tested for colorfastness at washed with water and with soap water solution of the dyed material following standard procedures given in [49]. The UV-Vis spectra recording and monitored the absorption at λ = 561 nm corresponding to the analytical signal of the peptide derivatives on the solutions after washing of the fabric, immediately and after 1 and 24 h were carried out. The soap solutions were pre-diluted twice with d.H_2_O and filtered through a paper filter to measure the absorbed radiation.

### 3.4. Virology

#### 3.4.1. Cytotoxicity Assay

Inoculation of monolayer cells in 96-well plates (Costar**^®^**, Corning Inc., Kennebunk, ME, USA) was performed with 0.1 mL/well-containing concentrations of the compounds diluted in a maintenance medium. Cells were incubated in a humidified atmosphere at 37 °C and 5% CO_2_ for 48 h. After microscopic evaluation, the maintenance medium containing the test compound was removed, cells were washed, and 0.1 mL of maintenance medium supplemented with 0.005% neutral red dye was added to each well, and cells were incubated at 37 °C for 3 h. After incubation, the neutral red day was removed, and cells were washed once with PBS, and 0.15 mL/well desorb solution (1% glacial acetic acid and 49% ethanol in distilled water) was added. The optical density (OD) of each well was read at 540 nm in a microplate reader (Biotek Organon, West Chester, PA, USA). The 50% cytotoxic concentration (CC_50_) was defined as the material concentration that reduced the cell viability by 50% when compared to untreated control.

#### 3.4.2. Antiviral Activity Assay

Antiviral screening was based on the viral yield reduction technique. Cytopathic effect (CPE) inhibition test used confluent cell monolayer in 96-well plates infected with 100 CCID_50_ in 0.1 mL. After 1 h of virus adsorption, the extract was added in various concentrations and cells were incubated for 48 h at 37 °C. The viable cells were stained according to the neutral red uptake procedure and the percentage of CPE inhibition for each concentration of the test sample was calculated using the following formula: % CPE = [OD_test sample_ − OD_virus control_]/[OD_toxicity control_ − OD_virus control_] × 100, where OD_test sample_ is the mean value of the ODs of the wells inoculated with virus and treated with the test sample in the respective concentration, OD_virus control_ is the mean value of the ODs of the virus control wells (with no compound in the medium) and OD_toxicity control_ is the mean value of the ODs of the wells not inoculated with virus but treated with the corresponding concentration of the test sample. The 50% inhibitory concentration (IC_50_) was defined as the concentration of the material that inhibited 50% of viral replication when compared to the virus control. The selectivity index (SI) was calculated from the ratio CC_50_/IC_50_.

#### 3.4.3. Virucidal Assay

Samples of 1 mL, containing human respiratory syncytial virus (HRSV-S2) (105,3 CCID50) or respectively human adenovirus type 5 (HAdV5) (106,3 CCID50), and tested compound in its maximum tolerable concentration (MTC) were contacted in a 1:1 ratio and subsequently stored at room temperature for different time intervals (30 and 60 min). Then, the residual infectious virus content in each sample was determined by the end-point dilution method, and Δlgs as compared to the untreated controls were evaluated.

Our approach to this test for virocidal effect of fabrics dyed with Rh-1, Rh-2 and Rh-3 was to cut identical pieces of the textiles (1 cm^2^) and immerse them in a viral suspension (100 µL) for the respective times (30 and 60 min). We used non dyed textiles of the same material for the control. The virus suspension was recovered by exhaustion after the appropriate time has elapsed. The residual infectious virus content was determined by the end-point dilution method followed by evaluation of Δlgs through comparison between each sample and control.

## 4. Conclusions

A systematic study of three novel rhodamine-peptide derivatives for textile dyeing has been presented. A modified with chloroacetyl chloride cotton fabric was used, which facilitated the deposition and retention of the new compounds. An intense and even color with good moisture resistance is obtained. A slight difference in the color characteristics of the tissues was found by colorimetric analysis. By using combined experimental approaches, the structural-textile application has been investigated by UV-Vis and fluorometric methods. Antiviral and virucidal activities of both the peptide-rhodamine B compounds and the dyed textile material were also studied. The most potent is Rh-3 peptide analogue, which is an analogue of hemorphin-4 containing a rhodamine B residue at the N-terminus and a hydrophobic -γ-Abu-Tyr-Pro-Trp-Thr-CONH_2_ amino acid sequence of the peptide molecule. Spectral studies have shown that the color of the aqueous solutions of compounds depends on their structural behavior and the type of solvent. In a polar protic solvent such as water, regardless of the pH of the medium, the compounds showed stability of ring-opened form and fluorescence on of the compounds without being affected by the number of methylene groups in the structure of the peptide moiety. The steric effect is more pronounced in the aprotic solvent of the compounds and the effect depends on the type of peptide chain. These structural characteristics are directly related to the dyeing of textile materials.

## Figures and Tables

**Table 1:** Physicochemical characterization of Rh-1, Rh-2, and Rh-3.

Compound	pK		pI	k, s^−1^	τ_1/2_
	pK_1_	pK_2_			
Rh-1	2.81	6.6	4.7	4.65 × 10^−2^ ± 0.0046	14.9
Rh-2	2.78	6.38	4.58	5.53 × 10^−2^ ± 0.0012	12.5
Rh-3	2.86	6.39	4.63	8.12 × 10^−2^ ± 0.0014	8.54

**Table 2:** Virucidal effect of new rhodamine B-conjugated hemorphin-4 analogues against human respiratory syncytial virus (HRSV-S2) and Human adenovirus serotype 5 (HAdV-5) after 30 min/60 min.

Virus	Δlog 30 min			Δlog 60 min		
	Rh-1	Rh-2	Rh-3	Rh-1	Rh-2	Rh-3
**HRSV-2**	0.2	0.4	0.2	1.3	1.3	1.7
**HAdV-5**	0	0	0	0	0	0

**Table 3:** Virucidal effect of new cotton fabrics dyed with rhodamine-peptides against human respiratory syncytial virus (HRSV-S2) and human adenovirus C serotype 5 (HAdV-5) after 30 min/60 min.

Virus	Δlog 30 min			Δlog 60 min		
	Rh-1-Textile	Rh-2-Textile	Rh-3-Textile	Rh-1-Textile	Rh-2-Textile	Rh-3-Textile
**HRSV-2**	−	−	−	0.2	0.2	0.1
**HAdV-5**	0	0	0	0	0	0

**Table 4:** Cytotoxicity and antiviral activity of new rhodamine-peptides against human respiratory syncytial virus (HRSV-S2) and Human adenovirus C serotype 5 (HAdV-5) in HEp-2 cell culture.

Compound	Cytotoxicity	Antiviral Activity			
	CC_50_ (µM/mL) in HEp-2 Cells	HRSV-S2		HAdV-5	
		IC_50_ (µM/mL)	SI	IC_50_ (µM/mL)	SI
Rh-1	35	NA	-	NA	-
Rh-2	23	NA	-	NA	-
Rh-3	113	NA	-	NA	-

## Data Availability

Data is contained within the article or Supplementary Material.

## Supplementary Materials

The following are available online, Figure S1. ESI-MS spectrum of Rh-1; Figure S2. ESI-MS spectrum of Rh-2; Figure S3. ESI-MS spectrum of Rh-3.

## Sample Availability

Samples of the compounds are available from the authors.
