# Supplementary material for: Synthesis of New Modified with Rhodamine B Peptides for Antiviral Protection of Textile Materials
Source: Molecules. 2021 Oct 31;26(21):6608. doi: 10.3390/molecules26216608 (PMC8587962; doi:10.3390/molecules26216608)
Supplement: Supplementary file 1 [file molecules-26-06608-s001.zip › Supplementary Material.pdf]

Supplementary Material

Synthesis of New Modified with Rhodamine B Peptides for Antiviral Protection of Textile Materials

Petar Todorov [1],* Stela Georgieva [2], Desislava Staneva [3], Petia Peneva [1], Petar Grozdanov [4], Ivanka Nikolova [4] and Ivo Grabchev [5]

[1] Department of Organic Chemistry, University of Chemical Technology and Metallurgy, 1756 Sofia, Bulgaria; e-mail: pepi_37@abv.bg
[2] Department of Analytical Chemistry, University of Chemical Technology and Metallurgy, 1756 Sofia, Bulgaria; e-mail: st.georgieva@uctm.edu
[3] Department of Textile and Leathers, University of Chemical Technology and Metallurgy, 1756 Sofia, Bulgaria; e-mail: grabcheva@mail.bg
[4] The Stephan Angeloff Institute of Microbiology, Bulgarian Academy of Sciences, 1113 Sofia, Bulgaria; e-mail: grozdanov@microbio.bas.bg
[5] Sofia University "St. Kl. Ohridski", Faculty of Medicine, 1407 Sofia, Bulgaria; e-mail: i.grabchev@chem.uni-sofia.bg
* Correspondence: pepi_37@abv.bg

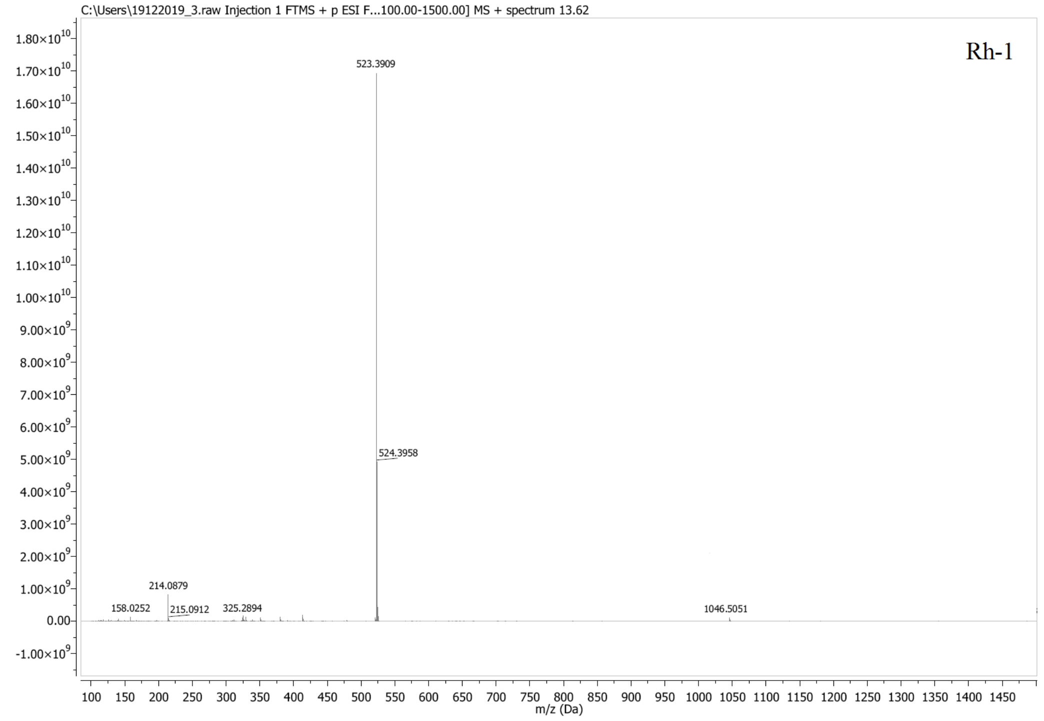

Figure **S1**. *ESI-MS spectrum of* ***Rh-1***

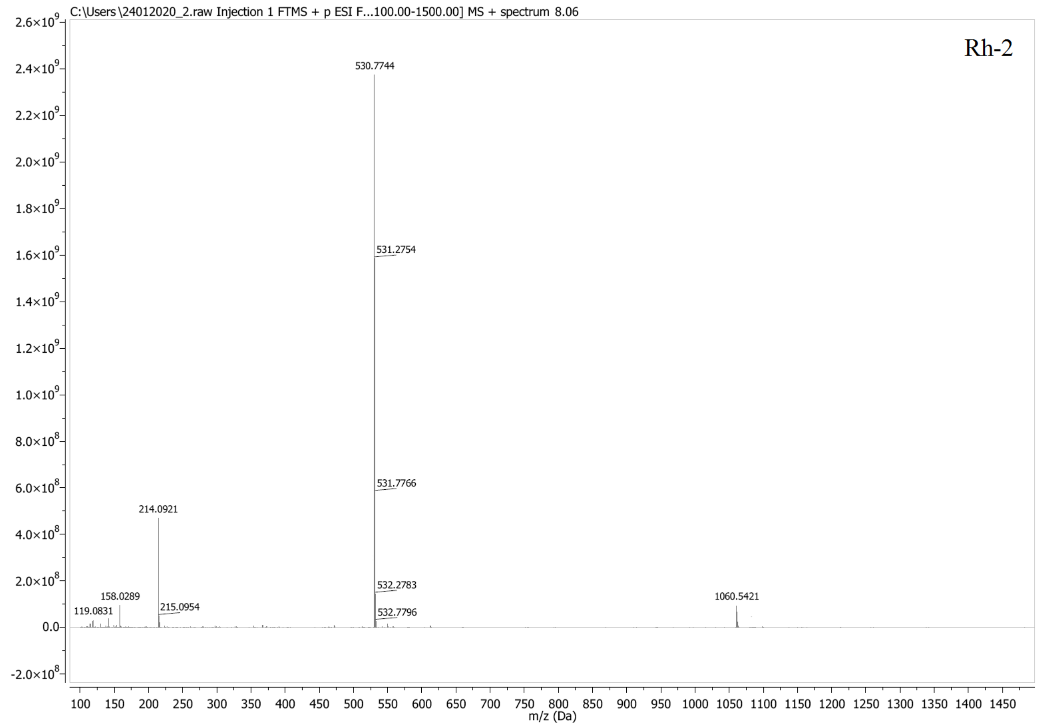

Figure **S2**. *ESI-MS spectrum of **Rh-2***

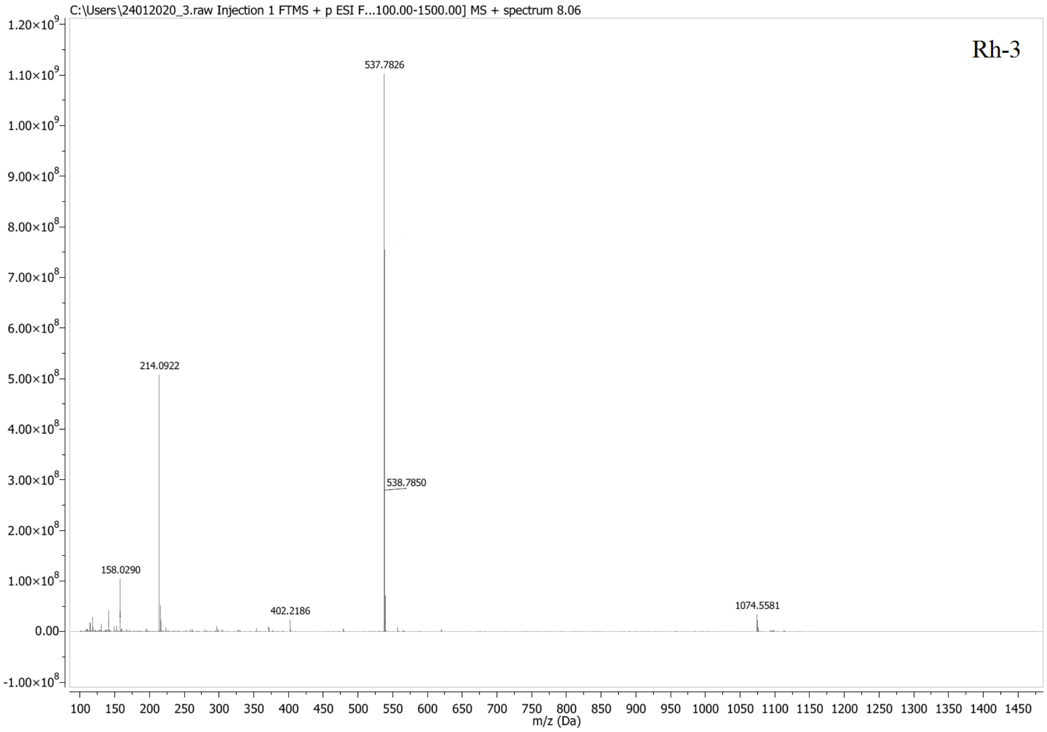

Figure **S3**. *ESI-MS spectrum of **Rh-3***
